# Notes and News

**Published:** 1898-03-19

**Authors:** 


### NOTES AND NEWS.

(Collected and Communicated.)

### HOSPITAL MEETINGS.

Metropolitan Hospital.—Annual Meeting, Tuesday, March 29th, 1.45 p.m.
Chelsea Hospital for Women.—Annual Meeting, Monday, March 21st, at 3 p.m., at the Hospital, Fulham Road.
Royal London Ophthalmic Hospital.—Annual Meeting, Tuesday, March 22nd, 12 noon, at the Hospital.
West End Hospital.—Annual Meeting, Friday, March 25th, 4 p.m., at the Hospital.
Royal Orthopædic Hospital.—Annual Meeting, Wednesday, 30th inst., at the Hospital.

Dr. J. H. Bryant has been appointed an assistant physician to Guy's Hospital.

The Lord Mayor is to give a banquet on May 4th, at the Mansion House, to the Presidents of the Royal Colleges of Physicians and Surgeons and other members of the medical profession.

In fiction the functions of the physician are frequently described in a manner amusing to the initiated. In a recent novel a scarlet fever patient in an aristocratic private hospital has ice "sent in to her at regular intervals by her own doctor."

On Mrs. Mona Caird's complaint that the London School Board has authorised a text-book on physiology for the use of pupil teachers which describes painful experiments on living animals, a committee has been appointed to report on the matter.

The Home for the Dying in Dublin, which accommodates 100 patients, is one of the most beautiful institutions of the kind in the world. Its mortuary is open to the public, is fitted like a chapel, and at all times people may be found there saying prayers for the dead.

Cerebro-spinal meningitis is said to have become epidemic among the gold-seekers in Alaska. The well-known fatality of this disease makes the news, if true, a matter for some anxiety. There can be no doubt that the cold, the hardship, and the want of food to which the miners are exposed, together with the overcrowding and neglect of sanitary precautions, which are sure to be rampant where labour is so dear as it is reported to be in Dawson City and at Klondyke, will greatly predispose the population to succumb to such an epidemic. It should be added that it is the general experience that this disease is most prone to occur in the winter and the spring.

Dr. W. J. Simpson, D.P.H., late health officer of Calcutta, has been appointed Professor of Hygiene in King's College, London.

The Queen has given her approval of the appointment of Professor John Glaister, M.D., of St. Mungo's College, Glasgow, to the vacant chair of Forensic Medicine in the University of Glasgow.

At the meeting of the Metropolitan Asylums Board held last Saturday a letter was received from the Local Government Board authorising the managers to erect a nurses' home at the Northern Hospital, and to expend for that purpose, together with additional work on the isolation block, a sum not exceeding £19,000.

It is not without interest to note that while we are troubling about the legalisation of the midwives, our colleagues at the other side of the Atlantic are being threatened from another quarter by the bone-setters, a Bill having been introduced into the New York States Legislature entitled "An Act regulating and legalising the practice of osteopathy." A similar Bill was recently introduced in Illinois, but was defeated owing to the protests of the medical profession.

Dr. Edward Noel Nason has lately been making some investigations as to the comparative prevalence of malignant disease in different districts which have given results of considerable interest. It is at once apparent that if one is to discover anything about the influence of the physical conditions of the different parts of the country in determining the varying prevalence of cancerous diseases very small areas must be dealt with, and this is what Dr. Nason has done. The result is to show very remarkable variations in the frequency of these diseases, differences apparently running side by side with differences of level, of drainage, and especially of nearness to sluggish rivers. His statistics, in fact, seem to indicate that cases of malignant diseases tend to group themselves chiefly about the low-lying land in the neighbourhood of sluggish streams, or where there is little fall and where the subsoil must in consequence be but indifferently drained. It would seem also that the closer to the stream the more frequent is the occurrence of the disease.

Last Monday the funeral took place of the late Mr. George Müller, of Bristol. Mr. Müller, who was in his 93rd year at the time of his death, was a very remarkable man and had had a remarkable career. It is now more than sixty years since he founded the Orphan Houses at Bristol in connection with which his name is so well known, homes which now have accommodation for 2,050 children. Mr. Müller is believed to have started his orphanage and for a long time to have carried it on without any open appeal to the public for assistance, and to have maintained an unshaken confidence that God would provide if He approved of the work that was being done. Many tales used to be current of the straits to which the homes were sometimes reduced, and of relief coming at the last moment in a "providential" manner from unexpected quarters, stories which, however good as illustrating the power of faith, did not speak well for the business capacity of providence. We fancy, however, that of recent years greater trust has been placed in printer's ink and ordinary methods of management.

